# A Rare Pituitary Tumor

**DOI:** 10.7759/cureus.63264

**Published:** 2024-06-27

**Authors:** Ramya Bhat, Nikhil Shankar, Chirag LU, Rakshith Srinivasa, Shilpa Rao, Pramila Kalra

**Affiliations:** 1 Internal Medicine, Mathikere Sampige (M S) Ramaiah Medical College Hospital, Bangalore, IND; 2 Endocrinology, Diabetes and Metabolism, Ramaiah Medical College, Bangalore, IND; 3 Neurosurgery, Mathikere Sampige (M S) Ramaiah Medical College Hospital, Bangalore, IND; 4 Neuropathology, National Institute of Mental Health & Neurosciences, Bangalore, IND; 5 Endocrinology and Metabolism, Mathikere Sampige (M S) Ramaiah Medical College Hospital, Bangalore, IND

**Keywords:** rare tumor, pituitary dysfunction, endocrinology, pituitary, supra sellar mass, pituitary tumour pituicytoma

## Abstract

Sellar-suprasellar masses, with diverse origins ranging from infiltrative to neoplastic processes, are frequently encountered in endocrinology clinics. Evaluation involves a detailed history, hormone analysis, and imaging of the hypothalamic-pituitary axis. However, overlapping hormonal and imaging features can complicate diagnosis, often necessitating confirmation through tissue biopsy. Pituicytoma, a rare sellar tumor mimicking other masses biochemically and radiologically, exemplifies this challenge. These are benign intracranial neoplasms with characteristic bipolar spindle-shaped astrocytic cells organized in fascicular or storiform patterns with specific immunohistochemistry. The current case is of an elderly postmenopausal woman with a history of hypertension who presented with recurrent headaches and transient vision loss in the left eye. Imaging studies revealed a suprasellar mass, which was biopsied and diagnosed on histopathological examination as a pituicytoma. This case highlights the importance of considering less common etiologies when encountering such presentations.

## Introduction

Given the paucity of documented sellar pituicytoma cases, numbering approximately 140 to date [1], this report offers a valuable contribution to the understanding of this rare neoplasm. Sellar masses, which can be pituitary adenomas, craniopharyngiomas, aneurysms, astrocytomas, and meningiomas, are often diagnosed with neuroimaging and hormone evaluation [2]. However, these tests may not be sufficient to distinguish between the different types. In some cases, a biopsy is needed along with biochemical testing for a definitive diagnosis. The patient in this case presented with headaches and vomiting and later developed diabetes insipidus.

## Case presentation

An elderly, postmenopausal lady with a history of hypertension presented to the endocrinology OPD complaining of a two-month history of headaches and vomiting. She also reported a transient loss of vision in her left eye lasting three days, which resolved spontaneously.

The patient had been experiencing recurrent dull, aching headaches over the past two months. The pain was intermittent, holocranial, and non-radiating; it was temporarily relieved by paracetamol but not entirely alleviated. Associated symptoms included non-projectile, non-bilious vomiting with food particles.

On examination, she was oriented to time, place, and person, with intact higher mental functions and a normal cranial nerve and motor system examination. There were no signs of sensory deficits or meningeal irritation. Otorhinological examination revealed a deviated nasal septum to the right, while ophthalmological evaluation showed normal findings. Visual field testing via perimetry was also normal, and other systemic examinations were unremarkable.

An MRI of the brain was performed due to persistent headaches, revealing an enlarged sella with a 16 × 17 × 19 mm lesion containing solid and cystic components, consistent with a pituitary macroadenoma. Coronal T1 images demonstrated an isointense lesion in the sellar and suprasellar regions with bilateral indentation of the diaphragm sella. Sagittal postcontrast T1 images showed heterogeneous enhancement of the sellar and suprasellar lesions, resulting in the expansion of the sella turcica. Axial images revealed a FLAIR hyperintense lesion in the sellar region. A surgical excision of the tumor was planned.

Prior to surgery, hormonal testing indicated low follicle-stimulating hormone and luteinizing hormone levels, with elevated prolactin levels. Other pituitary hormone levels were within normal limits (Table 1).

**Table 1:** Hormonal assay of the patient FSH, follicle-stimulating hormone; GH, growth hormone; LH, luteinizing hormone; PRL, prolactin; TSH, thyroid-stimulating hormone

Hormonal analyses	Patient value	Reference range
PRL (ng/ml)	63.2	3.8-23.6
Cortisol 8 AM (ug/dl)	12.93	5-23
FSH (mIU/ml)	4.56	25.8-134.8
LH (mIU/ml)	1.43	7.7-58.5
TSH (µIU/ml)	0.608	0.35-4.5
T3 (ng/dl)	100.4	40-181
T4 (µg/ml)	7	68-205
GH (ng/ml)	0.371	0.126-9.88

Her serum electrolytes, complete blood count, liver function tests, and renal function tests were all within normal limits (Table 2).

**Table 2:** Hemogram and biochemical parameters of the patient ALT, alanine aminotransferase; APTT, activated partial thromboplastin time; AST, aspartate aminotransferase; ESR, erythrocyte sedimentation rate; PT, prothrombin time

Investigation	Patient value	Reference range
Hemoglobin (g/dl)	13.5	12-16
Total leucocyte count (cells/cumm^3^)	6,100	4,000-11,000
Platelet count (cells/cumm^3^)	192,000	150,000-400,000
ESR (mm/hr)	9	1-23
Serum creatinine (mg/dl)	0.91	0.6-1.2
Serum sodium (mmol/l)	138	135-148
Serum potassium (mmol/l)	4.55	3.5-5.3
Serum calcium (mg/dl)	9.6	8.8-10.2
Serum phosphorous (mg/dl)	5.5	2.5-4.5
SGOT (U/L)	21	0-33
SGPT (U/L)	22	0-33
PT (seconds)	13.2	11.94-14.46
APTT (seconds)	29.1	26.46-30.4

The patient underwent trans-nasal trans-sphenoidal tumor excision. Extracapsular dissection was attempted, but the cyst ruptured, releasing apoplexy fluid. The cyst wall was excised and sent for histopathological examination. Postoperatively, she developed diabetes insipidus, which was treated with vasopressin injections. She was discharged with oral desmopressin and thyroxine tablets.

Furthermore, histopathology revealed fragments of a low-grade neoplasm composed of spindle to round cells arranged in short fascicles, sheets, and around blood vessels. The cells exhibited mild atypia, with round to oval nuclei and inconspicuous nucleoli. An adjacent normal adenohypophysis with preserved architecture was also observed.

## Discussion

Pituicytoma, formerly known as infundibuloma, is an uncommon tumor arising from specialized glial cells within the sellar and suprasellar regions of the brain [3]. They are extremely rare, accounting for only about 0.07% of all sellar lesions [4]. These slow-growing, benign tumors (classified as WHO grade I) are characterized by prominent blood vessel development [3]. A definitive diagnosis of pituicytoma requires a microscopic examination (histopathology) of surgically removed tissue. Treatment typically involves a complete surgical resection of the tumor, with a low risk of recurrence after surgery.

Pituicytomas primarily affect adults, with an average age of diagnosis of around 46.9 years (ranging from seven to 83 years) [3]. There may be a slight bias toward males being diagnosed with this condition. The specific symptoms experienced by patients vary depending on the size and location of the tumor within the brain. Since these tumors exert pressure on surrounding structures, the symptoms are often a consequence of this localized mass effect. Common presenting signs include disturbances in vision and visual field, headaches, hormonal deficiencies (hypopituitarism), and various neurological dysfunctions [3].

Pituicytomas can disrupt hormone production, with hyperprolactinemia (25.4%), hypopituitarism (19.6%), low testosterone in men (9.8%), and diabetes insipidus (3.9%) being the most frequently observed hormonal abnormalities [5].

**Figure 1:**
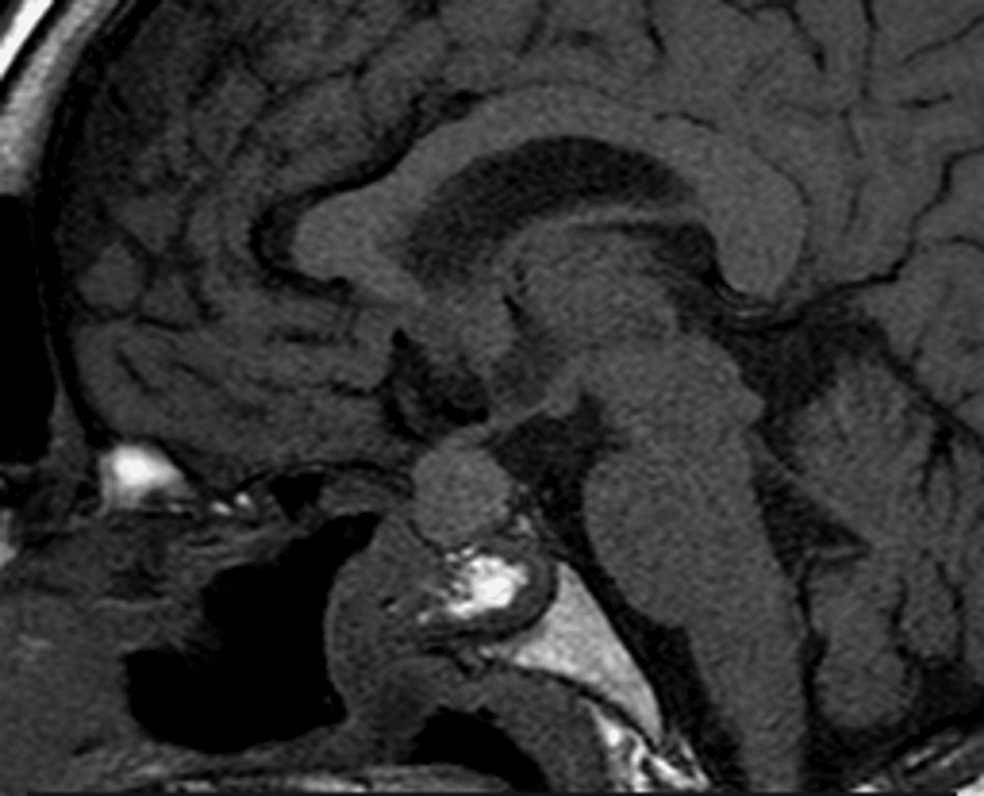
Saggital T1-weighted MRI image showing an isointense lesion in the sellar and suprasellar region

**Figure 2:**
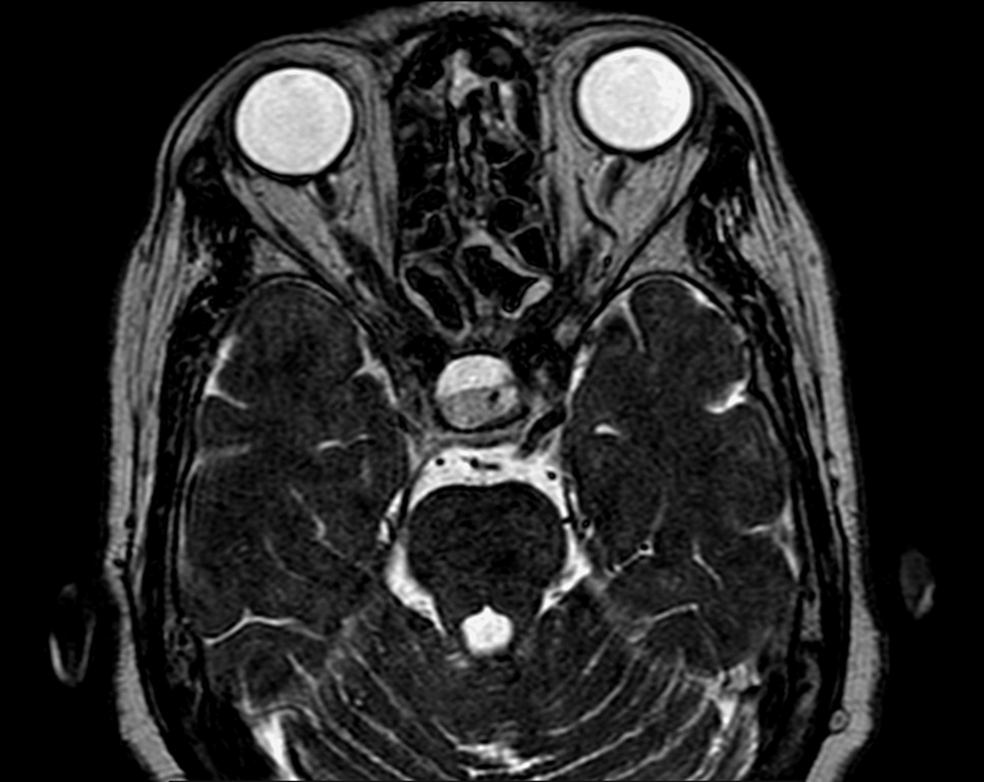
Axial image showing a FLAIR hyperintense lesion in the sellar region

**Figure 3:**
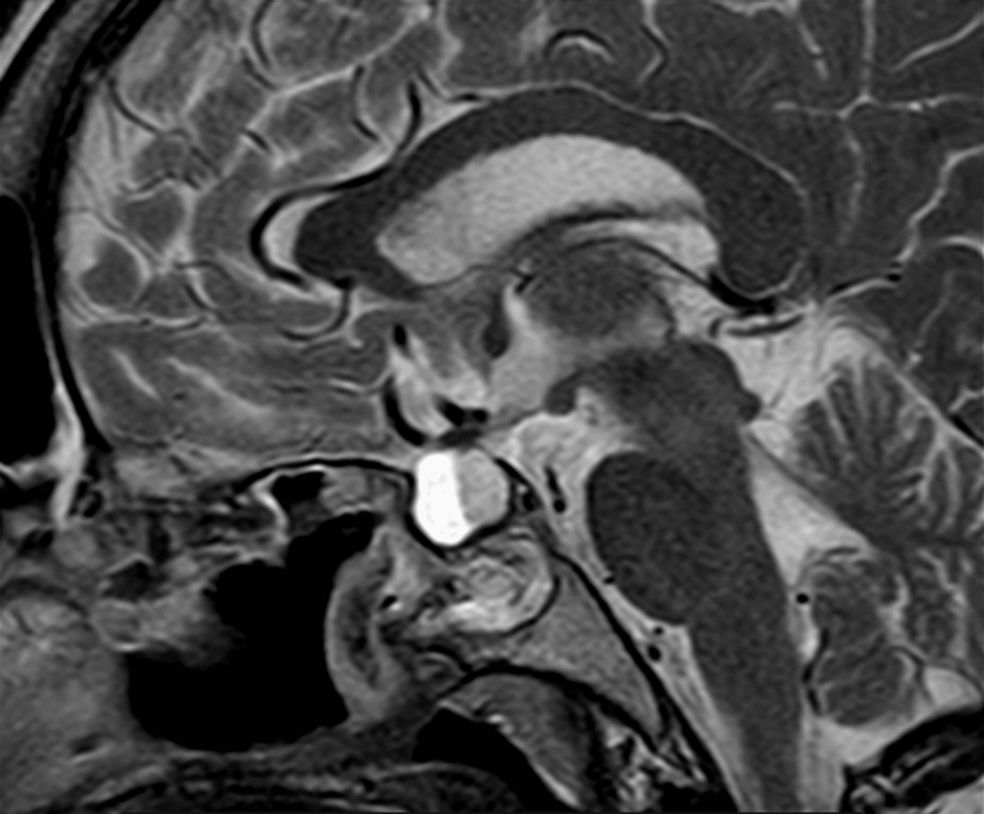
Sagittal postcontrast T2 image showing a heterogeneously enhanced sellar and suprasellar lesion causing expansion of sella turcica

While CT scans may show pituicytomas as varied-density masses in the sellar-suprasellar region with strong contrast uptake, MRI findings are less specific. On MRI, pituicytomas often appear as well-defined, solid masses within the sellar region, sometimes extending upward. Their signal intensity can vary, appearing low to similar to surrounding tissue on T1-weighted images and low to similar or slightly high on T2-weighted images. After contrast injection on T1-weighted images, they tend to show fairly uniform enhancement, which can closely resemble a suprasellar meningioma (Figure 1, Figure 2, Figure 3) [5].

**Figure 4:**
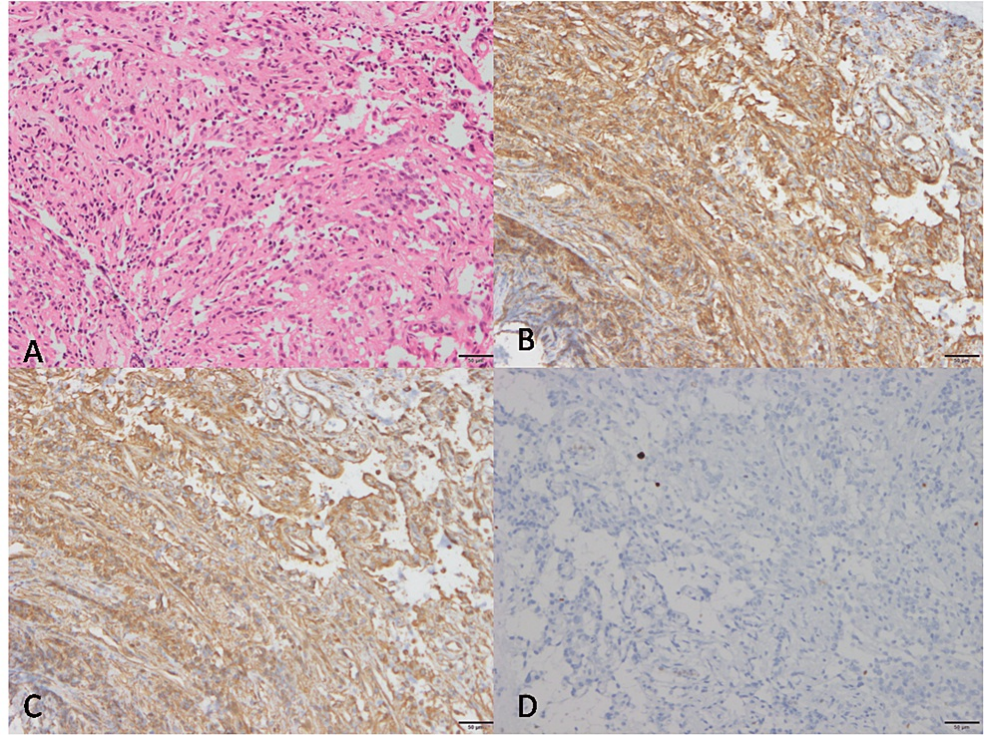
Short fascicles of spindled to polygonal cells (A, H&E), which are labeled by vimentin (B) and GFAP (C) and show low proliferation (D, MIB 1) GFAP, glial fibrillary acidic protein

Upon microscopic examination, this benign intracranial neoplasm exhibits a characteristic cell morphology originating from astrocytes. The tumor cells demonstrate a bipolar spindle-shaped morphology and are organized in fascicular or storiform patterns. The cytoplasm, the intracellular fluid, is abundant and eosinophilic, lacking granularity. Immunohistochemical analysis reveals a negative staining pattern for periodic acid-Schiff and the absence of Herring bodies, Rosenthal fibers, and EGBs, which are typically observed in normal neurohypophysis and pilocytic astrocytomas, respectively. While these tumor cells express S-100 and vimentin, their reactivity to glial fibrillary acidic protein is variable (Figure 4) [6].

We report a rare case of pituicytoma with clinical and endocrine abnormalities and immunohistochemical features consistent with those reported previously.

## Conclusions

This report describes a rare brain tumor and highlights the challenges of diagnosis. While imaging and hormone tests can identify a mass in the sellar and suprasellar regions, these tests might not provide a specific diagnosis. Pituicytomas are usually misdiagnosed as they show nonspecific clinical findings and radiological characteristics. Recognition of the lesion as a distinct entity is crucial for the correct diagnosis and treatment. Hormonal deficiency caused by pituicytoma is transient and needs periodic monitoring to avoid inadvertent hormone replacement.
